# Study on Syntaxonomic Diversity of Algal Cenoses in Soils of the Russian Far East, Using an Integrative Taxonomic Approach

**DOI:** 10.3390/plants13020306

**Published:** 2024-01-20

**Authors:** Shamil R. Abdullin, Arthur Yu. Nikulin, Veronika B. Bagmet, Vyacheslav Yu. Nikulin, Elena A. Zharikova, Irina V. Kiseleva, Andrey A. Gontcharov

**Affiliations:** Federal Scientific Center of the East Asia Terrestrial Biodiversity, Far Eastern Branch of the Russian Academy of Sciences, 159, 100-Letia Vladivostoka Prospect, Vladivostok 690022, Russia; artyrozz@mail.ru (A.Y.N.); chara1989@yandex.ru (V.B.B.); nikulinvyacheslav@gmail.com (V.Y.N.); ejarikova@mail.ru (E.A.Z.); kiseleva-iv@inbox.ru (I.V.K.); gontcharov@biosoil.ru (A.A.G.)

**Keywords:** soil algae, Braun-Blanquet approach, community, integrative approach, Russian Far East, PCA analysis, terrestrial habitats

## Abstract

Soil is a unique ecosystem with peculiar biodiversity that includes cyanobacteria and algae. Traditionally, cyanobacterial and algal cenoses were described mainly using the dominance approach, rarely based on the Braun-Blanquet method (floristic classification). More importantly, in both cases, the species of cyanobacteria and algae in communities were identified using classical methods (light microscopy) only. In this study, we present results of soil algal cenoses classification using the Braun-Blanquet approach based on species composition data obtained via an integrative approach. Characteristic tables include 19 out of 108 samples collected in the Jewish Autonomous Region, Primorsky Territory, and Sakhalin Region (Iturup Island) in 2018 and in 2020–2021. Twenty-five species of algae from four classes were identified in these sites. We described three new associations of algal communities—***Coelastrelletum aeroterrestricae*** ass. nova, ***Vischerietum magnae*** ass. nova, ***Bracteacoccetum bullati*** ass. nova. PCA analysis corroborated the results of syntaxonomic analysis and revealed that ***Coelastrelletum aeroterrestricae*** inhabit soils with a high value of P; ***Vischerietum magnae*** inhabit soils with high value of soil organic carbon (SOC), N, and higher humidity; and ***Bracteacoccetum bullati*** inhabit soils with high K values.

## 1. Introduction

Cyanobacteria and algae are important components of soil biota [1,2,3,4,5,6]. Specific life conditions in this habitat affect the species composition of these photosynthetic organisms, which sharply differs from those in aquatic environments. Cyanobacteria and algae represent an important resource for soil sustainable functioning [7,8,9,10]; therefore, the diversity of these organisms and their communities is under scrutiny in various habitats all over the world, e.g., as described in [11,12,13], and including Russia [14,15].

The species composition of freshwater cyanobacteria and algae has been well studied using classical methods in the Russian Far East [16,17]. However, information on their diversity in soils here is very scarce and based on sporadic collections only [18,19,20,21]. Moreover, these data, based on outdated methods and paradigms, do not reflect current levels of phycology development and require reevaluation from the standpoint of modern methods and approaches. The application of an integrative approach for the exploration of cyanobacteria and algae diversity in soils of this region has resulted in a description of new taxa and new distribution records [22,23,24,25]. Therefore, it could be expected that the use of this approach for studying the diversity of communities of cyanobacteria and algae may lead to the description of new syntaxa.

Previously cyanobacterial and algal cenoses were described mainly using the dominance approach. The floristic classification method differs due to the high information content of syntaxa (opportunities for bioindication), flexibility of classification criteria, openness of the system, successive nature of the classification development, consistent reductionism, use of the researcher’s intellectual potential, and a well-developed system of nomenclature [26]. That is why, nowadays, this approach is applied to classify macroalgae communities (MAC) and cyanobacterial–algal cenoses (CAC) (cyanobacterial (CC)/algal cenoses (AC)) in various ecosystems, ranging from the Atlantic Ocean and the Mediterranean Sea to soil algae in natural and disturbed environments [27,28,29,30,31,32]. Data on CAC (CC/AC) and MAC were summarized by Bültmann et al. [33] and Mucina et al. [12] who compiled high-ranked non-marine syntaxa of cyanobacteria and algae for the first Europe-wide checklist. These data comprised five classes of freshwater cyanobacteria and algae vegetation (***Charetea intermediate*** F. Fukarek 1961, ***Stigeoclonietea tenuis*** Arendt 1982, ***Lemaneetea fluviatilis*** Weber-Oldecop ex Bobrov et Chemeris 2012, ***Naviculetea gregariae*** Täuscher in Bültmann et al., 2015, ***Asterionelletea formosae*** Täuscher 1998), two classes of aerophytic cyanobacteria and algae vegetation (***Gloeocapsetea sanguineae*** Bültmann et Golubič in Bültmann et al., 2015, ***Desmococcetea olivacei*** Bültmann in Bültmann et al., 2015), one class of snow and ice algae vegetation (***Mesotaenietea berggrenii*** Bültmann et Takeuchi in Bültmann et al., 2015), and one class of soil cyanobacteria and algae vegetation (***Bracteacocco minoris–Hantzschietea amphioxyos*** Khaybullina et al., 2005). In addition to the Europe-wide checklist, a class of cave CAC (***Mychonastetea homosphaerae*** Abdullin 2020) was also described [29]. It should be mentioned that the species lists of cyanobacteria and algae, used to describe all these non-marine communities, were based on classical methods using only light microscopy. However, nowadays, the identification of algal species is mostly conducted using an integrative approach, including morphological studies based on light, confocal, transmission, and scanning electron microscopy, molecular genetic methods, analyses of the life cycle and sexual reproduction, etc. This approach allows us to accurately identify diagnostic species and the overall composition of communities that helps us to improve their classification.

Our work aimed to explore the syntaxonomical diversity of algal cenoses in soils of the Russian Far East using the Braun-Blanquet method, with algae species identified using the integrative approach. The latter was used for CAC (CC/AC) classification for the first time.

## 2. Results

### 2.1. Taxonomic Analysis of the Communities Described 

In the studied soil samples, 25 species of algae, belonging to four classes, were identified: Bacillariophyceae—2 spp.; Eustigmatophyceae—4 spp.; Chlorophyceae—14 spp.; and Trebouxiophyceae—5 spp.

### 2.2. Description of New Syntaxa

Based on the results of syntaxonomic analysis of the list of algal species revealed in the studied samples, we describe three new associations of algal communities without assigning them to any high-rank syntaxa.

Association ***Coelastrelletum aeroterrestricae*** Abdullin in Abdullin et al. ass. nova hoc loco. 

Holotypus: Sample 7 of Table 1 in this paper.

Diagnostic taxon: *Coelastrella aeroterrestrica* Tschaikner, Gärtner & Kofler (dom.).

Description: The association is a monodominant community of coccoid widespread green microalgae from the class Chlorophyceae. Four species of green algae and one species of eustigmatophytes were identified as sporadic (Table 1). The number of species in the sample varied from one to two. 

Ecology: Communities of this association are found in stony Haplic Cambisol, Haplic Cambisol, and Umbric Andosol from coniferous broad-leaved mixed forests and broad-leaved forests. Humidity in habitats varied from 29.4 to 78.9%, with a pH of 5.1–5.7, content of the soil organic carbon (SOC) of 4.1–6.6%, N of 0.7–1.3%, P of 4.8–24.0 mg/kg of soil, and K of 170.2–326.2 mg/kg of soil (Table 1). 

Distribution: Cenoses of the association are revealed in Primorsky Territory (Sedanka suburb of Vladivostok city, Shkotovsky, and Krasnoarmeisky Districts) and Sakhalin Region (Iturup Island).

Sporadic species: Sample 1: *Coelastrella terrestris* (Reisigl) Hegewald & N.Hanagata 1. Sample 2: *Chloromonas* cf. *reticulata* (Goroschankin) Gobi 1. Sample 3: *Vischeria vischeri* (Hibberd) Kryvenda, Rybalka, Wolf & Friedl 2. Sample 4: *Bracteacoccus minor* (Schmidle ex Chodat) Petrová 1. Sample 5: *Tetratostichococcus jenerensis* (Neustupa, Eliás & Sejnohová) Pröschold & Darienko 1. 

Sampling data: Sample 1—Russia, Primorsky Territory, Sedanka (suburb of Vladivostok city), 18 May 2021, 43°12′33.8″ N 131°59′57.7″ E. Sample 2—Russia, Primorsky Territory, Shkotovsky district, 23 June 2021, 43°34′54.8″ N 132°27′21.3”E. Sample 3—Russia, Sakhalin region, Iturup Island, 28 July 2018, 45°15′31.1″ N 147°55′10.9″ E. Sample 4—Russia, Primorsky Territory, Shkotovsky district, 23 June 2021, 43°34′54.2″ N 132°27′18.7″ E. Sample 5—Russia, Sakhalin region, Iturup Island, 27 July 2018, 45°12’30.4″ N 147°55′05.9″ E. Sample 6—Russia, Primorsky Territory, Krasnoarmeisky district, 15 July 2021, 45°57′37.1″ N 134°57′49.6″ E. Sample 7—Russia, Primorsky Territory, Shkotovsky district, 23 June 2021, 43°34′56.7″ N 132°27′19.6″ E. 

Samples’ collectors: Sample 1—A. Yu. Nikulin, V. Yu. Nikulin. Samples 2, 4, 7—A. Yu. Nikulin, R. Z. Allaguvatova. Samples 3, 5—E. M. Bulakh. Sample 6—Sh. R. Abdullin, A. Yu. Nikulin, V. Yu. Nikulin. 

Soil: Samples 1, 2, 4, 7—stony Haplic Cambisol. Samples 3, 5—Umbric Andosol. Sample 6—Haplic Cambisol. 

Vegetation: Samples 1, 2, 4, 6, 7—coniferous broad-leaved mixed forest. Samples 3, 5—broad-leaved forest. 

GenBank accession numbers: Sample 1: *Coelastrella aeroterrestrica* OQ873177. Sample 2: *Coelastrella aeroterrestrica* OQ873180; *Chloromonas* cf. *reticulata* OQ873167. Sample 3: *Coelastrella aeroterrestrica* OQ873178; *Vischeria vischeri* MW013808. Sample 4: *Coelastrella aeroterrestrica* OQ873176; *Bracteacoccus minor* OQ915508. Sample 5: *Coelastrella aeroterrestrica* OQ873179; *Tetratostichococcus jenerensis* OQ873173. Sample 6: *Coelastrella aeroterrestrica* OQ873174. Sample 7: *Coelastrella aeroterrestrica* OQ873175.

Association ***Vischerietum magnae*** Abdullin in Abdullin et al. ass. nova hoc loco. 

Holotypus: Sample 7 of Table 2 in this paper.

Diagnostic taxon: *Vischeria magna* (J.B.Petersen) Kryvenda, Rybalka, Wolf & Friedl (dom.).

Description: The association is a monodominant community of coccoid widespread microalgae from the class Eustigmatophyceae. Seven species of green algae, two species of diatoms, and two species of eustigmatophytes were identified as sporadic (Table 2). The number of species in the sample is 1–5. 

Ecology: Communities of association are described in stony Haplic Cambisol and Haplic Cambisol, Umbric Andosol, and Mollic Cambisol (Ornithic) from coniferous broad-leaved mixed forests, broad-leaved forests, oak forests, and weed meadow. Humidity in habitats varied from 44.1% to 79.3%, with a pH of 4.5–6.4, SOC of 3.9–11.6%, N of 0.9–2.1%, P of 0.4–27.9 mg/kg of soil, and K of 18.3–615.0 mg/kg of soil (Table 2).

Distribution: Cenoses of association are revealed in Primorsky Territory (Shkotovsky, Khasansky, and Krasnoarmeisky Districts, Furugelm Island) and Sakhalin Region (Iturup Island).

Sporadic species: Sample 1: *Chlamydomonas asymmetrica* Korshikov 1; *Coelastrella ellipsoidea* (P.M.Novis & G.Visnovksy) K.Gopalakrishnan, P.M.Novis & G.Visnovsky 1; *Coelastrella terrestris* (Reisigl) Hegewald & N.Hanagata 1; *Spongiochloris spongiosa* (Vischer) R.C.Starr 1. Sample 2: *Edaphochlorella mirabilis* (V.M.Andreyeva) Darienko & Pröschold in Darienko & al. 1; *Humidophila contenta* (Grunow) Lowe, Kociolek, J.R. Johansen, Van de Vijver, Lange-Bertalot & Kopalová 1; *Mayamaea arida* (Bock) Lange-Bertalot 1. Sample 3: *Monodopsis subterranea* (J.B.Petersen) D.J.Hibberd 1; *Parietochloris pseudoalveolaris* (T.R.Deason & Bold) Shin Watanabe & G.L.Floyd in Deason, Silva, Watanabe & Floyd 1. Sample 4: *Chloromonas* cf. *chlorococcoides* (H.Ettl & K.Schwarz) Matsukaki, Y.Hara & Nozaki 1. Sample 5: *Vischeria calaminaris* (Trzcinska et Pawlik-Skowronska) Kryvenda, Rybalka, Wolf & Friedl 1. 

Localities: Sample 1—Russia, Primorsky Territory, Shkotovsky district, 23 June 2021, 43°34′54.5″ N 132°27′22.3″ E. Sample 2—Russia, Sakhalin region, Iturup Island, 4 August 2018, 45°15′46.3″ N 147°57′32.2″ E. Sample 3—Russia, Sakhalin region, Iturup Island, 3 August 2018, 45°09′38.3″ N 147°46′39.7″ E. Sample 4—Russia, Primorsky Territory, Khasansky district, 29 July 2021, 42°47′26.8″ N 131°09′16.1″ E. Sample 5—Russia, Primorsky Territory, Khasansky district, Furugelm Island, 7 September 2018, 42°28′02.0″ N 130°55′34.0″ E. Sample 6—Russia, Primorsky Territory, Khasansky district, Furugelm Island, 7 September 2018, 42°27′34.0″ N 130°54′37.0″ E. Sample 7—Russia, Primorsky Territory, Krasnoarmeisky district, 15 July 2021, 45°57′36.5″ N 134°57′49.4″ E. 

Samples’ collectors: Sample 1—A. Yu. Nikulin, R. Z. Allaguvatova. Samples 2, 3—E. M. Bulakh. Sample 4—V. V. Shokhrina, R. Z. Allaguvatova. Samples 5, 6—Ye. A. Zharikova. Sample 7—Sh. R. Abdullin, A. Yu. Nikulin, V. Yu. Nikulin. 

Soil: Samples 1, 4—stony Haplic Cambisol. Samples 2, 3—Umbric Andosol. Sample 5—Mollic Cambisol (Ornithic). Samples 6, 7—Haplic Cambisol.

Vegetation: Samples 1, 3, 7—coniferous broad-leaved mixed forest. Samples 2, 6—broad-leaved forest. Sample 4—oak forest. Sample 5—weed meadow.

GenBank accession numbers: Sample 1: *Vischeria magna* OQ873181; *Coelastrella terrestris* OQ873193; *Chlamydomonas asymmetrica* OQ873195; *Spongiochloris spongiosa* OQ873168; *Coelastrella ellipsoidea* OQ873170. Sample 2: *Vischeria magna* OQ873183; *Humidophila contenta* OQ835556; *Edaphochlorella mirabilis* OQ873171; *Mayamaea arida* MZ400876. Sample 3: *Vischeria magna* OQ873182; *Monodopsis subterranea* MW013813; *Parietochloris pseudoalveolaris* MW013814. Sample 4: *Vischeria magna* OQ873186; *Chloromonas* cf. *chlorococcoides* OQ873191. Sample 5: *Vischeria magna* OQ873185; *Vischeria calaminaris* OQ873194. Sample 6: *Vischeria magna* OQ873184. Sample 7: *Vischeria magna* OQ873164.

Association ***Bracteacoccetum bullati*** Abdullin in Abdullin et al. ass. nova hoc loco. 

Holotypus: Sample 4 of Table 3 in this paper.

Diagnostic taxon: *Bracteacoccus bullatus* Fuciková, Flechtner & L.A.Lewis.

Description: The association is a community of coccoid microalgae from the class Chlorophyceae. In some samples, green microalgae *Heterochlamydomonas* cf. *callunae* (Ettl) Mikhailyuk & Demchenko was found. Five species of green algae were identified as sporadic (Table 3). The number of species in the sample varied from 2 to 3. 

Ecology: Communities of this association are found in stony Haplic Cambisol and Haplic Cambisol, and Umbric Andosol from coniferous broad-leaved mixed forests and grassland. Humidity in habitats varied from 49.1% to 77.4%, with a pH of 5.3–6.1, SOC of 4.2–5.9%, N of 0.7–1.5%, P of 3.5–11.3 mg/kg of soil, and K of 220.8–503.8 mg/kg of soil (Table 3).

Distribution: Cenoses of association are revealed in Primorsky Territory (Sedanka suburb of Vladivostok city), near the village of Kamenushka (Ussuriysk city district), Shkotovsky District), Jewish Autonomous Region (Obluchensky District, State natural reserve «Bastak»), and Sakhalin Region (Iturup Island).

Sporadic species: Sample 1: *Protosiphon botryoides* (Kütz.) Klebs 1. Sample 3: *Coccomyxa subellipsoidea* E.Acton 1; *Deuterostichococcus epilithicus* Pröschold & Darienko 1. Sample 4: *Coelastrella striolata* Chodat 1. Sample 5: *Chloromonas* sp. 1. 

Localities: Sample 1—Russia, Primorsky Territory, Shkotovsky district, 23 June 2021, 43°34′56.2″ N 132°27′18.6″ E. Sample 2—Russia, Primorsky Territory, Sedanka (suburb of Vladivostok city), 18 May 2021, 43°12′34.0″ N 131°59′59.2″ E. Sample 3—Russia, Sakhalin region, Iturup Island, 15 July 2022, 45°15′29.4″ N 148°10′34.0″ E. Sample 4—Russia, Primorsky Territory, near the village of Kamenushka (Ussuriysk city district), 6 June 2018, 43°36′29.3″ N 132°14′38.9″ E. Sample 5—Russia, Jewish Autonomous Region, Obluchensky district, State natural reserve «Bastak» 1 July 2021, 49°05′43.0″ N 133°04′56.3″ E.

Samples’ collectors: Sample 1—A. Yu. Nikulin, R. Z. Allaguvatova. Sample 2—A. Yu. Nikulin, V. Yu. Nikulin. Samples 3, 5—Sh. R. Abdullin, A. Yu. Nikulin. Sample 4—Sh. R. Abdullin. 

Soil: Samples 1, 2—stony Haplic Cambisol. Sample 3—Umbric Andosol. Samples 4, 5—Haplic Cambisol. 

Vegetation: Samples 1, 2, 4, 5—coniferous broad-leaved mixed forest. Sample 3—grassland. 

GenBank accession numbers: Sample 1: *Bracteacoccus bullatus* OQ873187; *Heterochlamydomonas* cf. *callunae* OQ915509; *Protosiphon botryoides* OQ873169. Sample 2: *Bracteacoccus bullatus* OQ873188; *Heterochlamydomonas* cf. *callunae* OQ915510. Sample 3: *Bracteacoccus bullatus* OQ873190; *Deuterostichococcus epilithicus* OQ873196; *Coccomyxa subellipsoidea* OQ873197. Sample 4: *Bracteacoccus bullatus* OQ873189; *Coelastrella striolata* OQ873192. Sample 5: *Bracteacoccus bullatus* OQ873165; *Chloromonas* sp. OQ873172.

### 2.3. Ordination Analysis

**Figure 1:**
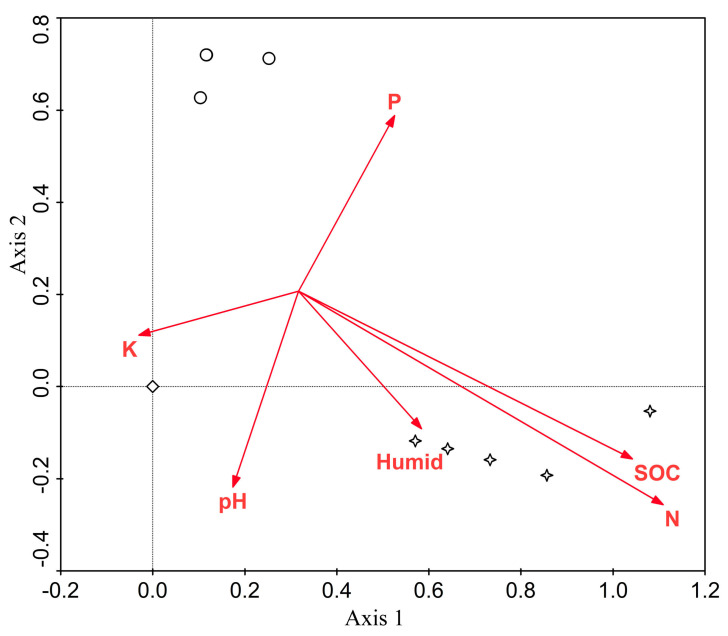
PCA biplot of algal communities in the soils of the Russian Far East, with axes 1 and 2 represented. Quantitative environmental variables are indicated by arrows. A (circles): association ***Coelastrelletum aeroterrestricae***; B (stars): association ***Vischerietum magnae***; C (diamond): association ***Bracteacoccetum bullati***. SOC—soil organic carbon; N—total nitrogen; Humid—humidity; pH—actual acidity; K—available potassium; P—available phosphorus. All 19 samples were included in the PCA biplot, but only 9 of them are visible because they are overlaying each other.

The PCA biplot shows three principal groups of AC, corresponding to three newly deduced associations (Table 1, Table 2 and Table 3) and their distinct separation according to abiotic factors (Figure 1; eigenvalues: axis 1—0.213; axis 2—0.184). The extreme position on the right on the first axis was occupied by ass. ***Vischerietum magnae*** with the highest values of SOC, N, and humidity in soil, whereas the upper position of the second axis was occupied by ass. ***Coelastrelletum aeroterrestricae*** with the highest value of P in soil. Ass. ***Bracteacoccetum bullati*** is situated at the intersection of the first and second axes with the highest value of K in soil. Ass. ***Coelastrelletum aeroterrestricae*** was found in more acidic habitats.

## 3. Discussion

Recently, it was suggested to limit syntaxa to communities formed by the macroscopic individuals within a plot, such as vascular plants, bryophytes, lichens, charophytes, and “macrophytic” chlorophytes, rhodophytes, and phaeophytes, because the sampling methods for microorganisms, including cyanobacteria and algae, are too different to produce results comparable with those of macroorganisms [34]. However, Theurillat et al. [35] point out that communities of microorganisms could be described and named within the Braun-Blanquet approach if these microorganisms are reliably identified using an integrative approach (including molecular genetic analysis), correctly named in accordance with the International Code of Nomenclature of Algae, Fungi and Plants (ICN; [36]), and these organisms at the sample site can be estimated by their frequency. The associations of microalgae ***Coelastrelletum aeroterrestricae***, ***Vischerietum magnae***, and ***Bracteacoccetum bullati*** that we described fully comply with these recommendations and are valid. The species composition of these communities was determined exclusively using an integrative approach. The sampling scale of the Braun-Blanquet approach was kept as simple as possible, allowing a valid description according to the ICPN (See Section 5), and communities were named in accordance with ICPN [35].

The new associations described in this study markedly differed from each other in composition and structure with only one species, *Coelastrella terrestris*, found in two associations, ***Coelastrelletum aeroterrestricae*** and ***Vischerietum magnae***. The association ***Vischerietum magnae*** was the most species-rich. Diatoms and three eustigmatophytes were found only in this sintaxon (Table 4). The ***Bracteacoccetum bullati*** was represented by green algae only (Table 4). The dominance of green algae (species of the class Chlorophyceae) was characteristic for all communities.

To date, only a few syntaxa of soil algae were described using the Braun-Blanquet approach, including one class, two orders, four alliances, ten associations, five subassociations, and two variants [14]. A comparative analysis of these and our syntaxa revealed that *Vischeria magna* (*Eustigmatos magnus* (J.B. Petersen) D.J. Hibberd) was among the diagnostic species of the order ***Eustigmatetalia magni*** Khaybullina et al., 2005 (class ***Bracteacocco minoris–Hantzschietea amphioxyos*** Khaibullina et al., 2005) [14]. The same species is diagnostic for our association ***Vischerietum magnae***. This, however, cannot be attributed to the latter order, since beside *Vischeria magna, Chlamydomonas globosa* J.W.Snow, *Cylindrospermum licheniforme* Kütz. ex Born. & Flah., *Hantzschia amphioxys* var. *capitata* Kant & P. Gupta, *Macrochloris dissecta* Korshikov, and *Coelastrella terrestris* (Reisigl) Hegewald & N. Hanagata (*Scotiellopsis terrestris* (Reisigl) Punc. & Kalina) are diagnostic species of the order ***Eustigmatetalia magni*** Khaybullina et al., 2005. Alliances and associations included in the order ***Eustigmatetalia magni*** Khaybullina et al., 2005 do not share diagnostic species with ***Vischerietum magnae***. Moreover, all diagnostic species characterizing the order ***Eustigmatetalia magni*** Khaybullina et al., 2005 were identified using light microscopy only, but not an integrative approach. When classifying in accordance with the Braun-Blanquet approach, in addition to the species composition, the ecotope and geographical location of the community are also taken into account [26]. Therefore, the difference in geographical location and soil type of our associations from the Russian Far East and previously described syntaxa from the Southern Urals is another argument confirming the distinctness of the new associations.

It is generally accepted that the new syntaxa at the rank of association or alliance could remain without subordination to higher syntaxa if they cannot be assigned to any of the previously identified higher syntaxa [26,35]. Therefore, we do not yet include the associations ***Coelastrelletum aeroterrestricae***, ***Vischerietum magnae***, and ***Bracteacoccetum bullati*** into any of the previously identified higher syntaxa. Additional studies are needed to describe the higher syntaxa. 

It was found that some edaphic factors influence the distribution of our syntaxa (Figure 1). When describing communities of microscopic cyanobacteria and algae in caves, the choice of a site for sampling was carried out within habitats with homogeneous environmental conditions [37], which made it possible to distinguish cenoses with the naked eye. This approach did not give a positive result during the study on communities of soil algae in the temperate monsoon climate zone of the Russian Far East. Apparently, this was due to the fact that the main factors influencing the distribution of cyanobacteria and algae communities in caves were the level of illumination and nature of moisture, which reflects the type of aquatic environment and flow velocity. Their gradation could be clearly distinguishable by the naked eye [37]. However, in the studied soil ecosystems, edaphic factors seem to play an important role in the distribution of algal communities, forming a more continuous habitat than in caves. In the future, this notion should be taken into account during the selection of sampling sites for a syntaxonomic analysis of cyanobacterial-algal communities in soils. In addition, it is necessary to conduct a parallel study of edaphic factors (content of macro- and microelements, humidity, pH, etc.) to clarify homogeneity. 

## 4. Conclusions

As a result of the successful combination of the integrative and Braun-Blanquet approaches, three associations of algae communities were described, for the first time, from soils of the temperate monsoon climate zone of the Russian Far East, ***Coelastrelletum aeroterrestricae***, ***Vischerietum magnae***, and ***Bracteacoccetum bullati***. In these syntaxa, 25 species of algae, belonging to four classes, were identified. Species of the class Chlorophyceae (Chlorophyta) dominated in all communities, while diatoms and three eustigmatophytes were found only in ***Vischerietum magnae***. New associations cannot be assigned yet to any high-rank syntaxa. PCA analysis showed three principal groups of algal cenoses, which corresponded to three newly deduced associations and the distinct separation of communities according to edaphic factors. It was revealed that ***Coelastrelletum aeroterrestricae*** inhabitat soils with a higher value of P, ***Vischerietum magnae*** inhabit soils with higher values of C and N and higher humidity, and ***Bracteacoccetum bullati*** inhabit soils with higher K values. Additional research is needed to resolve the methodological issue of choosing a soil sampling site for the syntaxonomic analysis of cyanobacteria and algae communities.

## 5. Materials and Methods

### 5.1. Study Site, Sampling, and Culture Conditions

The Russian Far East is the largest macro-region (over 40% of the Russian Federation), covering the basins of rivers flowing into the Pacific Ocean and the eastern part of the Arctic Ocean, as well as adjacent islands. Latitudinally, the Far East is located in five climatic zones. Part of this territory is located in the zone of a temperate monsoon climate, which covers both the mainland of the Far East (Primorsky and partly Khabarovsky Territories, south-east of Amur Territory and Jewish Autonomous Region), and island territories (Sakhalin Region) [38]. This type of climate is characterized by the predominance of cold air transfer from Eastern Siberia in winter, which causes cloudy dry weather with significant cold weather and a sharp minimum of precipitation. In summer, this area is dominated by cyclonic activity with heavy rainfall. Various forest communities dominate the vegetation of the Russian Far East. The soil-geographic zonation of Russia refers the southern part of the region to the coniferous broad-leaved mixed forests zone of Cambisols of the Eastern Cambisol forest region. Sakhalin and the Kuril Islands belong to the Far Eastern boreal-forest region [39].

**Figure 2:**
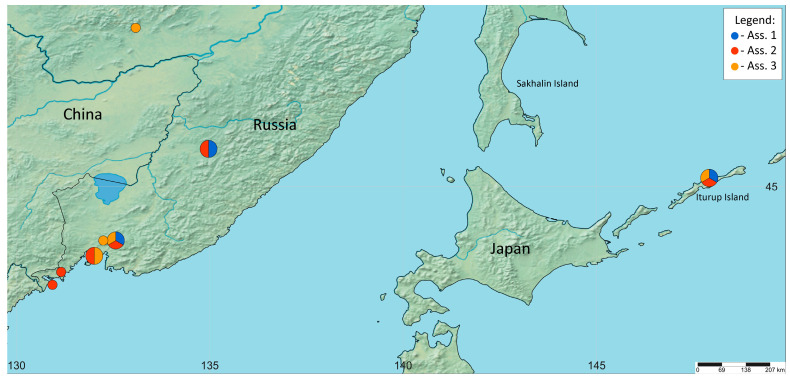
Map of the sample sites and association distribution. Association 1: ***Coelastrelletum aeroterrestricae***. Association 2: ***Vischerietum magnae***. Association 3: ***Bracteacoccetum bullati***.

This study is based on 108 soil samples collected from the Jewish Autonomous Region, Primorsky Territory, and Sakhalin Region (Iturup Island) (Figure 2, Table 1, Table 2 and Table 3) in 2018 and in 2020–2021. Characteristic tables include 19 samples. In general, AC could not be detected with the naked eye. Samples for identifying cyanobacteria and algae communities are analogous to relevés for identifying vascular plant communities, and the sampling of soils was carried out using standard methods [40] in volumes of 125 cm^3^ within habitats with homogeneous environmental conditions [37]. The species composition of the AC was identified using the integrative approach (i.e., the cultivation of samples in nutrient medium, strain isolation, light and scanning electron microscopy, and molecular genetic methods). The species abundance was measured via direct methods (light and scanning electron microscopy) and after cultivation. The sampling scale of the Braun-Blanquet approach was kept as simple as possible allowing a valid description, according to the ICPN [35], with a sampling scale of three degrees: 1—the species was identified once using direct methods or in liquid nutrient medium in the sample; 2—the species was identified 2–10 times using direct methods in the sample; 3—the species was identified more than 10 times using direct methods in the sample.

For identification of the species composition of algae in soils, the strains were isolated from the samples using the micropipette method [41] and cultured in liquid nutrient media Waris-H with silica [41,42] and 3N BBM [41] at 20–22 °C and an irradiance of 17.9–21.4 μmol photons m^−2^ s^−1^ with a 16:8 h (light/dark) photoperiod. The strains were maintained in the culture collection of the Laboratory of Botany in the Federal Scientific Center of East Asian Terrestrial Biodiversity, Russian Federation.

### 5.2. Light and Scanning Electron Microscopy, Preliminary Identification

The morphology of vegetative and reproductive cells of algae was examined using an Olympus BX 53 light microscope (LM; Olympus Corporation, Tokyo, Japan) equipped with Nomarski DIC optics and an Olympus DP27 digital camera (Olympus Corporation, Tokyo, Japan). Diatom frustules were cleaned via oxidation with hydrogen peroxide, rinsed several times with distilled water, and mounted in Elyashev medium [43] having a refractive index of 1.67–1.68. For scanning electron microscopy (SEM; Merlin, Carl Zeiss, Germany, Instrumental Centre of Biotechnology and Gene Engineering, FSCEATB FEB RAS), the material was dried onto brass stubs and coated with chrome. A number of identification keys were consulted [44,45,46,47,48,49,50,51,52,53]. The taxonomy of algae was chosen according to Guiry & Guiry [54]. 

### 5.3. DNA Extraction, PCR, Species Identification

Selected strains of algae were studied using molecular genetic methods. Cultures were harvested during the exponential growth phase and concentrated via centrifugation. Total genomic DNA was extracted as described previously [55]. For the Chlorophyta and Eustigmatophyceae members, 18S rDNA or the ITS rDNA region were amplified using primer combinations and temperature profiles, following López-García et al. [56] and Marin et al. [57]. The amplification and sequencing of the chloroplast *rbc*L gene of Bacillariophyta were conducted as described previously [58].

PCR amplification was performed using the Encyclo Plus PCR kit (Evrogen, Moscow, Russia) with a T100 Thermal Cycler (Bio-Rad Laboratories, Inc., Hercules, CA, USA). The PCR products were purified by ExoSAP-IT PCR Product Cleanup Reagent (Affymetrix Inc., Santa Clara, CA, USA) and sequenced in both directions at the Instrumental Centre of Biotechnology and Gene Engineering of Federal Scientific Centre of the East Asia Terrestrial Biodiversity FEB RAS using an ABI 3500 genetic analyzer (Applied Biosystems, Foster City, CA, USA) with a BigDye terminator v. 3.1 sequencing kit (Applied Biosystems, Waltham, MA, USA). The 18S and ITS rDNA PCR products overlapped for ca. 300 bp, which ensured a non-chimeric concatenated sequence. Sequences were assembled with the Staden Package v.1.4 [59], deposited into the GenBank under the accession numbers provided in Table S1.

After a morphological examination of the strains, their sequences were compared with those from strains available at the National Center for Biotechnology Information (NCBI, Bethesda, MD, USA) via a BLAST search (https://blast.ncbi.nlm.nih.gov/Blast.cgi; accessed on 6 June 2023) for clarification of their morphology-based identification. For each strain, the most similar sequence was determined not only by the percentage of identity but also by its phylogenetic position among closely related sequences. It was checked using the “Distance tree of results” option (fast minimum evolution and neighbor joining methods) implemented in BLAST. In the case of 98–100% similarity with sequences from the NCBI, identity at the species level was assumed. However, due to unresolved placement in the phylogenetic trees, or high percentage of identity to apparently misidentified algae, for some specimens, tentative species name (cf.) or only generic assignment was given. The BLAST results are provided in Table S1.

### 5.4. Syntaxonomical Analysis and Ordination

The classification of cyanobacteria and algae communities followed the Braun-Blanquet approach [60,61].

Ordination analysis was performed using Canoco 4.5/CanoDraw 4.0 software [62]. The structure of the dataset was tested with detrended correspondence analysis (DCA), and the gradient length of the first DCA axis (1 SD units) indicated an application of linear ordination techniques. Principal component analysis (PCA), considering axes 1 and 2, was performed to detect the main environmental factors affecting the species composition of the sites in question and to visualize any differences between them. Default options included focus scaling on inter-sample distances, species scores divided by standard deviation, and centering by samples without transformation of the species data applied in PCA. The PCA biplot was used to illustrate the main gradients of soil data in the floristic composition of algae communities. 

Syntaxon names follow the International Code of Phytosociological Nomenclature [35].

### 5.5. Soil Analysis

Soil humidity (Humid) was determined via the thermostatic weight method by drying samples at 105 °C [63].

The content of the soil organic carbon (SOC) was determined using the wet combustion method of Tyurin, which is very close to the Walkey–Black method [63]. This method is included in the register of methods approved for the determination of organic matter in soils of the Russian Federation [64]. According to the GOST 26213-91 guidelines, the SOC was extracted with a mixture of K_2_Cr_2_O_7_ and concentrated H_2_SO_4_, followed by titration with 0.2 mol/L of Mohr’s salt. The content of nitrogen (N) in soils was determined using a Thermo Flash 2000 elemental analyzer (Thermo Fisher Scientific, Waltham, MA, USA).

The pH of water suspensions was measured via potentiometry using a pH meter, Mettler Toledo FiveEasy F20 (Mettler Toledo Greifensee, Switzerland). Briefly, available phosphorus (P) and available potassium (K) were extracted from the soil using hydrochloric acid solution (concentration 0.2 M). Then, P was determined quantitatively using a photometry method and KFK-2MP (ZOMZ, Sergiev Posad, Russia), and K was determined using a flame photometry method and PFA-378 (Unico-Sys, St. Petersburg, Russia) [63]. Each analysis was conducted in triplicate. Data are given as means. 

## Figures and Tables

**Table 1:** Association ***Coelastrelletum aeroterrestricae*** Abdullin in Abdullin et al. ass. nova hoc loco.

No.	1	2	3	4	5	6	7 *	Constancy
Humidity, %	78.9	68.3	59.2	67.8	55.2	29.4	51.3	
pH	5.7	5.2	5.4	5.1	5.4	5.4	5.6	
SOC, %	4.1	6.6	5.3	6.1	5.3	5.5	4.3	
N, %	0.7	1.3	1.1	1.3	1.1	1.0	1.0	
P, mg/kg of soil	4.8	19.2	15.3	24.0	15.3	19.2	9.2	
K, mg/kg of soil	326.2	198.4	241.5	170.2	233.2	286.4	226.6	
No. of species	2	2	2	2	2	1	1	
Diagnostic species of the ass. ***Coelastrelletum aeroterrestricae***								
*Coelastrella aeroterrestrica* Tschaikner, Gärtner & Kofler	3	3	3	3	3	3	3	V^3^

Notes. *—holotypus.

**Table 2:** Association ***Vischerietum magnae*** Abdullin in Abdullin et al. ass. nova hoc loco.

No.	1	2	3	4	5	6	7 *	Constancy
Humidity, %	73.9	65.2	66.0	79.3	65.5	66.5	44.1	
pH	4.5	5.5	5.7	5.6	6.4	5.5	6.3	
SOC, %	11.6	7.4	7.5	5.5	5.1	3.9	10.7	
N, %	2.1	1.4	1.6	0.9	1.6	1.4	1.5	
P, mg/kg of soil	27.9	12.2	11.8	5.7	2.6	0.4	25.7	
K, mg/kg of soil	247.3	214.1	215.0	176.0	19.1	18.3	615.0	
No. of species	5	4	3	2	2	1	1	
Diagnostic species of the ass. ***Vischerietum magnae***								
*Vischeria magna* (J.B. Petersen) Kryvenda, Rybalka, Wolf & Friedl	3	3	3	3	3	2	2	V^2–3^

Notes. *—holotypus.

**Table 3:** Association ***Bracteacoccetum bullati*** Abdullin in Abdullin et al. ass. nova hoc loco.

No.	1	2	3	4 *	5	Constancy
Humidity, %	68.3	49.1	77.4	65.3	66.4	
pH	5.5	6.0	6.1	5.7	5.3	
SOC, %	4.2	4.4	5.4	5.0	5.9	
N, %	0.9	0.7	1.0	1.0	1.5	
P, mg/kg of soil	10.0	5.2	3.5	7.4	11.3	
K, mg/kg of soil	220.8	503.8	368.5	372.7	398.4	
No. of species	3	2	3	2	2	
Diagnostic species of the ass. ***Bracteacoccetum bullati***						
*Bracteacoccus bullatus* Fuciková, Flechtner & L.A.Lewis	3	3	3	3	3	V^3^
Other species						
*Heterochlamydomonas* cf. *callunae* (Ettl) Mikhailyuk & Demchenko	1	1	.	.	.	II

Notes *—holotypus.

**Table 4:** Taxonomic composition of the cenoses described in the soils of the Russian Far East.

Class	Associations		
	1	2	3
Bacillariophyceae	0	2	0
Eustigmatophyceae	1	3	0
Chlorophyceae	4	5	5
Trebouxiophyceae	1	2	2
Total number of species, by AC	6	12	7
Total number of samples, by AC	7	7	5

Notes: 1—association ***Coelastrelletum aeroterrestricae***; 2—association ***Vischerietum magnae***; 3—association ***Bracteacoccetum bullati***.

## Data Availability

The data presented in this study are available on request from the corresponding author. In addition, the data that support the findings of this study are openly available in GenBank.

## Supplementary Materials

The following supporting information can be downloaded at https://www.mdpi.com/article/10.3390/plants13020306/s1, Table S1: Results of genotyping of the isolated algal strains from soils using the BLAST algorithm.
