# Supplementary material for: Study on Syntaxonomic Diversity of Algal Cenoses in Soils of the Russian Far East, Using an Integrative Taxonomic Approach
Source: Plants (Basel). 2024 Jan 20;13(2):306. doi: 10.3390/plants13020306 (PMC10819320; doi:10.3390/plants13020306)
Supplement: Supplementary file 1 [file plants-13-00306-s001.zip › Table S1.pdf]

Study on syntaxonomic diversity of algal cenoses in soils of the Russian Far East, using integrative taxonomic approach

Shamil R. Abdullin*, Arthur Yu. Nikulin, Veronika B. Bagmet, Vyacheslav Yu. Nikulin, Elena A. Zharikova, Irina V. Kiseleva, and Andrey A. Gontcharov

Table S1. Results of genotyping of the isolated algal strains from soils using the BLAST algorithm.

Taxa	Strain	GenBank accession number	Phylogenetic DNA region	Percentage of identity with the closest strain from NCBI
Coelastrella aeroterrestrica Tschaikner, Gärtner & Kofler	VCA-240 Ch	OQ873174	ITS rDNA	100.00% – *Coelastrella aeroterrestrica* SWK1_2 JX513879
	VCA-227 Ch	OQ873175	ITS rDNA	100.00% – *Coelastrella aeroterrestrica* IT2-2-2 MW013807
	VCA-231 Ch	OQ873176	ITS rDNA	100.00% – *Coelastrella aeroterrestrica* HP-29P MZ969857
	VCA-221 Ch	OQ873177	ITS rDNA	99.29% – *Coelastrella aeroterrestrica* Ru-1-8 MH703752
	VCA-34 Ch	OQ873178	ITS rDNA	100.00% – *Coelastrella aeroterrestrica* SWK1_2 JX513879
	VCA-32 Ch	OQ873179	ITS rDNA	99.88% – *Coelastrella aeroterrestrica* SWK1_2 JX513879
	VCA-225 Ch	OQ873180	ITS rDNA	100.00% – *Coelastrella aeroterrestrica* SWK1_2 JX513879
Vischeria magna (Petersen) Kryvenda, Rybalka, Wolf & Friedl	VCA-233 Eu	OQ873181	ITS rDNA	98.63% – *Eustigmatos magnus* SAG 2554 MG596348
	VCA-27 Eu	OQ873182	ITS rDNA	100.00% – *Vischeria magna* ACSSI 013 MK228872
	VCA-29 Eu	OQ873183	ITS rDNA	98,88% – *Eustigmatos magnus* SAG 2554 MG596348
	VCA-218 Eu	OQ873184	ITS rDNA	99.76% – *Eustigmatos magnus* SAG 2554 MG596348

	VCA-219 Eu<br>VCA-241 Eu<br>VCA-242 Eu	OQ873185<br>OQ873164<br>OQ873186	ITS rDNA<br>18S rDNA<br>ITS rDNA	99.76% – *Eustigmatos magnus* SAG 2554 MG596348<br>100.00% – *Vischeria magna* ACSSI 081 MK228873<br>99.09% – *Vischeria magna* SAG 2554 MG596348
*Heterochlamydomona*s cf. *callunae* (Ettl) Mikhailyuk & Demchenko	VCA-229 Ch<br>VCA-224 Ch	OQ915509<br>OQ915510	18S rDNA<br>18S rDNA	99.75% – *Chlamydomonas callunae* Us-1-5 MH703756<br>99.75% – *Chlamydomonas callunae* Us-1-5 MH703756
Bracteacoccus bullatus Fuciková, Flechtner & L.A.Lewis	VCA-228 Ch<br>VCA-223 Ch<br>VCA-238 Ch<br>VCA-216 Ch<br>VCA-244 Ch	OQ873187<br>OQ873188<br>OQ873165<br>OQ873189<br>OQ873190	ITS rDNA<br>ITS rDNA<br>18S rDNA<br>ITS rDNA<br>ITS rDNA	99.57% – *Bracteacoccus bullatus* SAG 2032 JQ281848<br>99.57% – *Bracteacoccus bullatus* SAG 2032 JQ281848<br>100.00% – *Bracteacoccus bullatus* ACSSI MG582205<br>99.64% – *Bracteacoccus bullatus* MZ-Ch11 KY066480<br>99.34% – *Bracteacoccus bullatus* SAG 2032 MW075321
Humidophila contenta (Grunow) Lowe, Kociolek, J.R. Johansen, Van de Vijver, Lange-Bertalot & Kopalová	VCA-214 Ba	OQ835556*	*rbc*L	93.21% – *Pinnularia* cf. *gibba* 12 EF143304
Chloromonas cf. *chlorococcoides* (H.Ettl & K.Schwarz) Matsukaki, Y.Hara & Nozaki	VCA-243 Ch	OQ873191	ITS rDNA	98.83% – *Chloromonas chlorococcoides* SAG72.81 AB624573
Coelastrella striolata Chodat	VCA-217 Ch	OQ873192	ITS rDNA	100% – *Coelastrella striolata* ACSSI 355 MZ920145
Coelastrella terrestris (Reisigl) Hegewald & N.Hanagata	VCA-222 Ch<br>VCA-234 Ch	OQ873166<br>OQ873193	18S rDNA<br>ITS rDNA	99.27% – *Coelastrella terrestris* IRK-A 173 MK504637<br>99.53% – *Coelastrella terrestris* IRK-A MK504637
Chloromonas cf. *reticulata* (Goroschankin) Gobi	VCA-226 Ch	OQ873167	18S rDNA	98.49% – *Chloromonas reticulata* ARK-S12-19 OM729982
Edaphochlorella mirabilis (V.M.Andreyeva) Darienko & Pröschold in Darienko & al.	VCA-215 Ch	OQ873171	18S rDNA	100% – *Edaphochlorella mirabilis* ACSSI 102 MG696560
Mayamaea arida (Bock) Lange-Bertalot	VCA-44 Ba	MZ400876*	*rbc*L	94.84% – *Mayamaea terrestris* NIES-4281 LC648449
Vischeria calaminaris (Trzcinska et Pawlik-Skowronska) Kryvenda, Rybalka, Wolf & Friedl	VCA-220 Eu	OQ873194	ITS rDNA	99.72% – *Vischeria calaminaris* E120 JX202554
Vischeria vischeri (Hibberd) Kryvenda, Rybalka, Wolf & Friedl	VCA-30 Eu	MW013808	ITS rDNA	99.88% – *Eustigmatos vischeri* CCAP 860/7 KJ713283
Monodopsis subterranea (J.B.Petersen) D.J.Hibberd	VCA-28 Eu	MW013813	ITS rDNA	99,69% – *Monodopsis subterranea* U41054
Chlamydomonas asymmetrica Korshikov	VCA-235 Ch	OQ873195	ITS rDNA	99.81% – *Chlamydomonas asymmetrica* A2WS ON077054
Chloromonas sp.	VCA-239 Ch	OQ873172	18S rDNA	99.75% – *Chloromonas* sp. ACSSI 397 OQ363207

Spongiochloris spongiosa (Vischer) R.C.Starr	VCA-236 Ch	OQ873168	18S rDNA	99.90% – *Spongiochloris spongiosa* CCAP 3/1 MK541715
Protosiphon botryoides (Kützing) Klebs	VCA-230 Ch	OQ873169	18S rDNA	100.00% – *Protosiphon botryoides* GTD4b-3 KC149964
Bracteacoccus minor (Schmidle ex Chodat) Petrová	VCA-232 Ch	OQ915508	18S rDNA	99.07% – *Bracteacoccus minor* SAG 221-1 MW075319
Coelastrella ellipsoidea (P.M.Novis & G.Visnovksy) K.Gopalakrishnan, P.M.Novis & G.Visnovsky	VCA-237 Ch	OQ873170	18S rDNA	99.62% – *Coelastrella ellipsoidea* LCR-CG7 KC861672
Parietochloris pseudoalveolaris (T.R.Deason & Bold) Shin Watanabe & G.L.Floyd in Deason, Silva, Watanabe & Floyd	VCA-38 Ch	MW013814	ITS rDNA	99.84% – *Ettlia pseudoalveolaris* NV-5 MT735204
Tetratostichococcus jenerensis (Neustupa, Eliás & Sejnohová) Pröschold & Darienko	VCA-213 Ch	OQ873173	18S rDNA	100.00% – *Tetratostichococcus jenerensis* KP09HW3001 KX094816
Deuterostichococcus epilithicus Pröschold & Darienko	VCA-245 Ch	OQ873196	ITS rDNA	98.8% – *Deuterostichococcus epilithicus* SAG 2060 MT07816
Coccomyxa subellipsoidea E.Acton	VCA-246 Ch	OQ873197	ITS rDNA	99.56% – *Coccomyxa subellipsoidea* C-169 FAHX137-B20 AC277064

Notes: * – represent the sequences of new species for the NCBI.
